# Analgesia Quality and Duration of Fentanyl and Sufentanil Added to Bupivacaine in Spinal Anesthesia in Inguinal Hernia Surgery: A Double‐Blind Parallel Randomized Controlled Trial

**DOI:** 10.1002/hsr2.71116

**Published:** 2025-07-27

**Authors:** Haniyeh Soleimany, Hamed Tavan, Ebrahim Khalighi, Reza Pakzad

**Affiliations:** ^1^ Student Research Committee Ilam University of Medical Sciences Ilam Iran; ^2^ Faculty of Nursing and Midwifery Ilam University of Medical Sciences Ilam Iran; ^3^ Department of Anesthesiology, School of Allied Medical Sciences Ilam University of Medical Sciences Ilam Iran; ^4^ Health and Environment Research Center Ilam University of Medical Sciences Ilam Iran; ^5^ Department of Epidemiology, Faculty of Health Ilam University of Medical Sciences Ilam Iran

**Keywords:** Bupivacaine, Fentanyl, inguinal hernia surgery, pain, randomized clinical trial, Sufentanil

## Abstract

**Background and Aims:**

To compare the quality and duration of analgesia provided by Fentanyl and Sufentanil when added to 12–15 mg Bupivacaine 0.5% in spinal anesthesia for inguinal hernia surgery.

**Methods:**

In the present double‐blind parallel Randomized Controlled Trial (RCT), 180 patients who were candidates for elective inguinal hernia surgery were divided into three groups using balanced‐block randomization. The first group received 2.5 cc Bupivacaine with 0.5 cc (25 mg) Fentanyl, the second group received 0.5 cc (2.5 micrograms) of Sufentanil with 2.5 cc Bupivacaine, and the third group received 2.5 cc Bupivacaine with 0.5 cc normal saline. The pain score was evaluated by VAS (visual analog scale) at recovery and arrival in PACU, 2, 4, 6, and 12 h after the surgery.

**Results:**

A total of 60 subjects were allocated to each group. The mean (standard deviation) of pain score 2 h after surgery in Bupivacaine, Bupivacaine‐Fentanyl, and Bupivacaine‐Sufentanil were 1.73 (0.49), 1.10 (1.10), and 1.22 (1.25), respectively. These values 12 h after surgery were 2.76 (0.36), 2.97 (0.61), and 2.92 (0.56), respectively. The within‐group comparison showed a significant increase in the pain score over time (*p* < 0.001). Also, the between‐group comparison revealed significant differences between the three groups regarding pain scores (*p* = 0.030), so that the mean and standard deviation of pain score in Bupivacaine (1.67 ± 0.05) was higher than Bupivacaine‐Fentanyl (1.51 ± 0.09) (*p* < 0.001) and Bupivacaine‐Sufentanil (1.52 ± 0.08) (*p* < 0.001), but Bupivacaine‐Fentanyl was not different from Bupivacaine‐Sufentanil (*p* = 0.437). Results showed a significant interaction between time and intervention for pain score (*p* < 0.001).

**Conclusions:**

The pain‐reducing effects of Sufentanil and Fentanyl, when combined with Bupivacaine, were significantly greater than those of Bupivacaine alone. However, this effect lasts only for up to 4 h after surgery, after which their efficacy diminishes.

## 1 Introduction

One of the most common elective procedures performed globally is the repair of an inguinal hernia [1], which is performed annually on more than 20 million people [2]. Moderate to severe pain following inguinal hernia repair is frequently reported, and it may result in an extended hospital stay. Additionally, inadequate postoperative pain management has been identified as a risk factor for persistent chronic pain following inguinal hernia repair [3]. Various surgical and anesthetic methods are utilized to treat inguinal hernia [4], with the choice of anesthetic technique depending on factors such as patient and surgeon preference, anesthesiologist's experience, suitability for the individual patient, pain control, early recovery, and post‐operative costs [5].

General, regional, and local anesthesia methods facilitate open inguinal hernia surgery [2, 6]. Many studies aim to identify the benefits of these three anesthetic methods for inguinal hernia repair [7]. An ideal anesthesia technique provides adequate analgesia before, during, and after the operation (perioperative), has minimal complications, facilitates early patient discharge, and is cost‐effective [2]. Spinal anesthesia (SA) is a popular method for inguinal hernia repair [6], offering advantages such as simplicity, rapid onset, and consistent sensory and motor blocks [8, 9, 10]. Additionally, SA can provide sufficient analgesia during and immediately after surgery at a lower cost [11]. Currently, the most common drug used for SA is Bupivacaine [12], which acts by blocking voltage‐gated sodium channels.

Bupivacaine is an amide local anesthetic known for its prolonged duration of effect and low incidence of transient radicular symptoms. However, a high dose intrathecal Bupivacaine is more likely to produce a high spinal block with upper limb paralysis, loss of consciousness, and apnea [13, 14]. Despite its efficacy, Bupivacaine may sometimes fail to alleviate visceral pain and discomfort resulting from peritoneal stretching [15]. To address these limitations, clinicians often add a small amount of opioids to the local anesthetic solution, leading to faster onset, reducing the required dose of local anesthetics, improving intraoperative anesthesia quality, and extending the postoperative pain‐free period [10, 12, 15, 16, 17, 18, 19, 20, 21]. Nevertheless, intrathecal opioids may lead to some side effects, such as nausea, vomiting, itching, and respiratory depression [17]. Opioids synergize with Bupivacaine, enhancing anesthesia and its duration [10, 12]. Lipophilic opioids like Fentanyl and Sufentanil are particularly suitable for SA due to their pharmacological properties, improving analgesia both during and after surgery [9, 10, 19]. In other words, Bupivacaine acts on Na channels on the nerve membranes while lipophilic opioids act on specific receptors in the posterior horn of the spinal cord and have a small systemic effect by absorption.

Ultimately, providing pain‐free surgery and a pain‐free postoperative period is perhaps one of the greatest gifts for patients. Despite numerous studies examining the effects of various opioids combined with anesthetic drugs in different surgeries, it appears that no study has specifically investigated this issue in inguinal hernia repair surgery. This study aims to compare the effects of Fentanyl and Sufentanil combined with Bupivacaine on the quality and duration of analgesia following inguinal hernia repair surgery using SA.

## 2 Method

### 2.1 Design, Setting, and Participants

This was a three‐arm (with 1:1 allocation ratio) Double‐Blind Parallel Randomized Controlled Trial (IRCT code: IRCTID: IRCT20240509061717N1) that was done on all male patients who were candidates for inguinal hernia repair surgery with SA referred to Imam Khomeini Hospital in Ilam City (located west of Iran) in 2024. The Full trial protocol can be accessed at: https://irct.behdasht.gov.ir/trial/76845. The report of the present study was according to Assel et al.'s [22] recommendation and Consolidated Standards of Reporting Trials (CONSORT) guideline, and their checklist was available in the Supporting Information S1: Appendix S1.

### 2.2 Sample Size

Based on a similar study [12], in which the pain scores 24 h after surgery, were 6.3 ± 1.9 in the Fentanyl group and 7.3 ± 2 in the Placebo group and using the formula below [23] to have *β* = 20%, *α* = 5%, the sample size was estimated to be 58 in each group.

$$n=2\times \;\frac{{\left(Z_{1-\alpha /2}+Z_{1-\beta }\right)}^{2}\;\times \;\left(S_{1}^{2}+S_{2}^{2}\right)}{{\left(\mu_{1}-\mu_{2}\right)}^{2}}=\frac{\;{\left(1.96+0.84\right)}^{2}\;\times \;(1.9^{2}+2^{2})}{{\left(6.3-7.3\right)}^{2}}=58.$$

A total of 5% was added to the calculated sample size to compensate for the power decline due to attrition, loss to follow‐up, and missing data. Finally, 61 samples were considered in each group (183 in total).

### 2.3 Inclusion and Exclusion Criteria

All adult male patients who were candidates for inguinal hernia repair surgery with ASA Grades I–II and aged 18–70 years were included. Patients with a BMI > 35 kg/m^2^ were excluded due to the possibility of failure in spinal technique, incomplete block, inability to bend the back, improper positioning, or inability to set the appropriate level when entering the needle. Also, Patients with dependence on or consumption of opioids, local infection at the injection site, back pain, blood coagulation disease, allergy to opioids and local anesthetics, and patients who needed general anesthesia were excluded. Also, if the patients required additional drugs, such as opioids/morphine and benzodiazepines, during the operation, they were excluded, too. Additionally, any patients who experienced significant side effects after intervention of surgery, such as severe vomiting or nausea, severe hypotension, or rare drug sensitivities like Transient neurological symptoms (due to its bad prognosis) [24] or other similar sensitivity, were excluded from the analysis.

### 2.4 Sampling and Random Assignment

**Figure 1:**
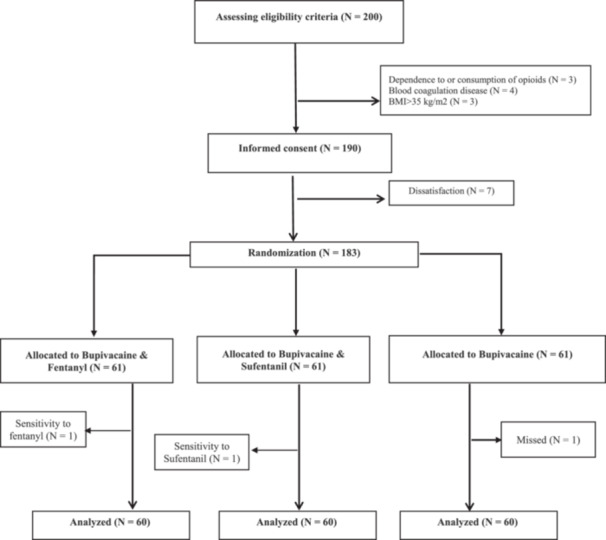
Flowchart of study design.

Two hundred patients were initially chosen to be hospitalized for surgery to correct an inguinal hernia. A total of 190 patients were left after the inclusion and exclusion criteria of the study were applied. The objectives of the study were thoroughly described to these patients, and their informed consent was obtained. Seven patients were removed from the trial after refusing to give their consent after being given the explanation (see Figure 1). Finally, 183 patients were assigned to the Bupivacaine, Bupivacaine with Fentanyl, and Bupivacaine with Sufentanil groups using balance‐block randomization in blocks of 6. The “alloc” package in Stata software was used to create the random blocks. H‐S enrolled the participants, and the methodologist's coauthor (R‐P) generated a random allocation sequence.

### 2.5 Concealment and Blinding

Balance‐block randomization method was used to keep both participants and researchers blinded to the treatment allocation. To achieve concealment, a series of encoded randomization envelopes was created based on a random allocation sequence, with each code indicating a distinct type of medication. The principal investigator (R‐P) opened the recruitment envelopes one by one, determining each participant's assignment using a prepared list of codes corresponding to the medications. Importantly, the code list was only accessible to the principal investigator, ensuring that the other coauthors remained blind to the patient's group assignment. Because patient assignments were concealed until the end of the statistical analysis, participants, the anesthesiologist or the person responsible for dispensing medicine, and the examiner were blind to the group allocations throughout the study.

### 2.6 Intervention

Patients at the entrance to the operating room received 5–7 mL/kg NaCl 0.9% intravenously, and their vital signs, including systolic blood pressure, diastolic blood pressure, electrocardiogram (ECG), heart rate, and blood oxygen saturation percentage, were monitored and recorded by a trained nurse. Then, SA was performed in the sitting position using a 25–27 spinal needle (25‐gauge Quincke needle, Mekon Medical Devices Co., Shanghai, China) in the L3‐L4 or L4‐L5 interspace. Patients in the first group received 0.5 cc (25 micrograms) of Fentanyl (Caspian Tamin Co., Rasht, Iran) plus 2.5 cc (12 mg) of Bupivacaine 0.5% (2.5 mL; AstraZeneca, Austria), patients in the second group received 0.5 cc (2.5 mg) of Sufentanil (Sufiject Aburaihan Co., Iran), plus 2.5 cc (12 mg) of Bupivacaine 0.5% and patients in the third group received 2.5 cc (12 mg) of Bupivacaine 0.5% plus 0.5 cc normal saline. It should be noted that the exact dose of drugs was determined according to the anesthesiologist's decision, and preparation of medicines for intrathecally injection was done by another anesthesiology technician (H‐T).

To do a sterile injection [25], the anesthesiology technician prepared the necessary equipment (including sterile gloves, sterile spinal sets, Betadine, spinal needles, 5 cc syringes, a Bupivacaine ampule, a Fentanyl ampule, a Sufentanil ampule, and sterile normal saline). The anesthesiology technician opened the first layer of the spinal set, and the person giving the injection (anesthesiologist) first put on a mask, then sterile gloves, and opened the second layer of the sterile set. The anesthesiology technician then placed the remaining equipment sterilely inside the spinal set. The anesthesiologist cleaned the back three times with Betadine after determining the injection site, dried the cleaned area, and, with the help of the anesthesiology technician, drew the injectable solution into the sterile syringe and performed the injection.

After the SA, the patient was placed in the supine position, and the level of sensory block was checked regularly with a pinprick test in the midclavicular line. The time of the sensory block reaching the level of T6 was determined as the time that the patient was ready for surgery. Also, the level of motor block was determined with the Bromage score [26] after SA. It should be noted that to determine the level of motor block, we defined a complete block (according to Bromage score) as when the patient was unable to move their foot or knee. Then, systolic blood pressure, diastolic blood pressure, ECG, heart rate, and blood oxygen saturation percentage will be controlled and recorded every 5 min until the end of the procedure.

It should be noted that several surgeons performed surgery using a unique approach. The surgery technique for all patients was Open Inguinal Hernia Repair [27], and after the end of the surgery, the patients were transferred to the recovery room.

### 2.7 Primary Outcome

A trained nurse (H‐S) evaluated Patients’ pain six times, including in the recovery unit, at arrival, 2, 4, 6, and 12 h after transfer to the surgical ward. The pain score was assessed by VAS (visual analog scale with a 0–10 scale) during the evaluation. If the patients had vomiting or nausea, antiemetic drugs were prescribed (Ondansetron or Metoclopramide, according to the patient's situation). Patients with a pain score of 3 or higher after surgery received a dose of intravenous morphine. The first instance of morphine administration, the total number of times morphine was given after the operation, and the duration of each patient's stay in recovery were all recorded.

### 2.8 Secondary Outcomes

Duration of surgery, duration of the spinal block, time to sensory block at T6, and the level of motor block were defined as some secondary outcomes.

### 2.9 Side Effects

In the present study, nausea, vomiting, pruritus, bradycardia, and hypotension were considered as intervention side effects. These side effects were measured after the operation and before they received postoperative morphine.

### 2.10 Statistical Analysis

Stata version 11 was used to analyze the data while taking the Intention‐to‐Treat [24] strategy into account. The data distribution's normality was evaluated using the Kolmogorov–Smirnov test. The baseline data was compared using the Student's *t*‐test. One‐way repeated measure ANOVA was used to compare the effect of the interventions at the six time points by adjusting for first‐time morphine injection and the total number of times that morphine was given. Also, the Bonferroni and least significant difference (LSD) tests were used for pairwise/multiple comparisons. Additionally, a one‐way ANOVA was employed to compare secondary outcomes, including surgery time, time to sensory block at T6, and time to motor block and if necessary, the Tukey post‐hoc test was applied for pairwise comparisons. Finally, the Chi‐squared/Fisher exact test was used to compare the side effects in study groups. Data are presented as mean and standard deviation (SD) for quantitative variables and number (%) for qualitative variables. The significance level was set at *p* < 0.05.

### 2.11 Ethical Considerations

The protocol of this project has been approved by the Ilam University of Medical Sciences, which has an ethics code (IR.MEDILAM.REC.1400.149). The study was conducted in accordance with the current Declaration of Helsinki, and informed consent was obtained from all individual participants.

## 3 Results

Given that after the random allocation of subjects in the study groups, two patients in the Fentanyl and Sufentanil groups experienced sensitivity to intervention (transient neurological symptoms) after surgery. Also, one patient in the Bupivacaine group was lost from follow‐up due to personal concerns. Finally, 60 patients were compared in each group. All the information was measured, and there was no missing data. All quantitative variables were normally distributed according to Kolmogorov–Smirnov test results (*p* > 0.05). The mean (SD) age of the subjects in Bupivacaine, Bupivacaine‐Fentanyl, and Bupivacaine‐Sufentanil was 54 (11.2), 57.9 (10.1), and 55.4 (10.2) years, respectively. The means (SD) of all variables showed no statistical differences and are shown in Table 1.

**Table 1:** Comparison of basic variables between the study groups^a^.

Variables	BUPI (*N* = 60)	BUPI‐FENT (*N* = 60)	BUPI‐SUF (*N* = 60)	*p* value
Hematological variables^a^				
WBC (10^3^/µL)	8.3 ± 3.1	7.6 ± 3.6	8.4 ± 3.6	0.459
RBC (10^6^/µL)	4.9 ± 1.2	4.9 ± 1.8	5.1 ± 1.3	0.834
Hb (g/dL)	12.4 ± 4.3	12.5 ± 4.7	12.4 ± 4.1	0.983
Hct (%)	34.7 ± 13.2	32.2 ± 16.2	35.1 ± 12.5	0.505
MCV (30)	79 ± 19.2	74.1 ± 24.4	78.6 ± 18.8	0.415
MCH (pg)	27.5 ± 6.4	25.6 ± 8.4	27.3 ± 6.8	0.312
MCHC (g/dL)	32.2 ± 7.7	30.5 ± 9.7	32.2 ± 7.8	0.467
Plt	261.7 ± 82.8	233.9 ± 68.9	239 ± 70.5	0.117
Neutrophil (%)	66.3 ± 18.2	65 ± 23.2	66.2 ± 19.1	0.940
Lymphocyte (%)	21.3 ± 14.1	16.8 ± 12.7	18.2 ± 13.7	0.208
PT (second)	13.9 ± 1.6	14.3 ± 1.4	14.2 ± 1.8	0.318
PTT (second)	33.3 ± 4.8	34.9 ± 4.3	34.2 ± 5	0.168
INR	1.1 ± 0.2	1.2 ± 0.2	1.2 ± 0.2	0.651
Liver function^a^				
AST (U/L)	56.8 ± 45.4	60.5 ± 52.9	51.1 ± 37.4	0.529
ALT (U/L)	63.8 ± 131	59.1 ± 47.9	66.9 ± 99.6	0.910
Renal function^a^				
Cr (mg/dL)	1.9 ± 0.8	1.9 ± 0.8	1.8 ± 0.7	0.548
BUN (mg/dL)	41.7 ± 35.7	38.3 ± 27.9	41.8 ± 38.2	0.814
Background variables^a^				
Age (yrs old)	54 ± 11.2	57.9 ± 10.1	55.4 ± 10.2	0.126
Height (m)	168.8 ± 9.9	167.4 ± 10.9	170.3 ± 9.4	0.286
Weight (Kg)	74 ± 10.7	72.8 ± 12.1	73.8 ± 12.9	0.858
BMI (Kg/m^2^)	26.2 ± 4.6	26 ± 3.9	25.5 ± 4.3	0.664
Morphine dosage^b^				
1 dose (5 mg)	4 (6.7)	41 (68.3)	34 (56.7)	< 0.001*
2 dose (10 mg)	20 (33.3)	19 (31.7)	25 (41.7)	
3 dose (15 mg)	36 (60)	0 (0)	1 (1.7)	
First time of morphine need (minute)^a^	147.75 ± 16.71	228.33 ± 19.37	220.33 ± 17.51	< 0.001*
Duration of the spinal block (minute)^a^	97 ± 10.82	161.25 ± 15.67	155.42 ± 15.22	< 0.001*

Abbreviations: BMI, body mass index; BUPI, Bupivacaine; BUPI‐FENT, Bupivacaine and Fentanyl; BUPI‐SUF, Bupivacaine and Sufentanil; Hb, hemoglobin; Hct, Hematocrit; INR, international normalized ratio; MCH, mean corpuscular hemoglobin; MCHC, mean corpuscular, hemoglobin concentration; MCV, mean corpuscular volume; PTT, prothrombin time test; RBC, red blood cell; WBC, white blood cell.

^a^ Quantitative variables were presented as mean ± standard deviation.

^b^ Qualitative variables were presented as numbers (%). Quantitative and qualitative variables were compared between the three groups using independent *t*‐test and Chi‐square test, respectively. The significance level was considered as 0.05.

### 3.1 Morphine Administration

As mentioned in the method section and as shown in Table 1, all patients received morphine after surgery. However, its dosage was different between the three groups. In Bupivacaine group, 20 (33.3%) patients received 2 doses (10 mg) of morphine, and 36 (60%) patients received 3 doses (15 mg) of morphine after surgery. However, in the Bupivacaine‐Fentanyl group, only 19 (31.7%) patients received 2 doses (10 mg) of morphine, and other (41 patients or 68.3%) received only 1 dose (5 mg) of opioid. In addition, none of them needed 3 doses (15 mg) of morphine after surgery. In Bupivacaine‐Sufentanil 25 (41.7%) patients received 2 doses (10 mg) of morphine, and only 1 patient (1.7%) received 3 doses (15 mg) of morphine after surgery. Results of the Chi‐squared test (*ꭓ* ^2^ = 98.473, degree of freedom: 4; *p* < 0.001) revealed that the distribution of morphine dosage was different between the three groups, and patients in the Bupivacaine group needed more morphine after surgery in comparison with Bupivacaine‐Fentanyl and Bupivacaine‐Sufentanil.

Furthermore, the result of the ANOVA test revealed that the mean (SD) of the first time that patients need to morphine in Bupivacaine group [147.75 (16.71)] was lower than Bupivacaine‐Fentanyl [228.33 (19.37)] and Bupivacaine‐Sufentanil [220.33 (17.51)] (*p* < 0.001).

### 3.2 Duration of the Spinal Block

The mean (SD) of the duration of the spinal block (minute) in Bupivacaine, Bupivacaine‐Fentanyl, and Bupivacaine‐Sufentanil was 97 (10.82), 161.25 (15.67), and 155.42 (15.22), respectively. The result of the ANOVA test revealed that the duration of the spinal block in Bupivacaine was lower than Bupivacaine‐Fentanyl and Bupivacaine‐Sufentanil (*F* statistic = 382.448; df = 2177; *p* < 0.001).

### 3.3 Analgesic Effect of Fentanyl and Sufentanil (Primary Outcome)

#### 3.3.1 Intra‐Participant Variability (Time Effect) in the Pain Scores

**Figure 2:**
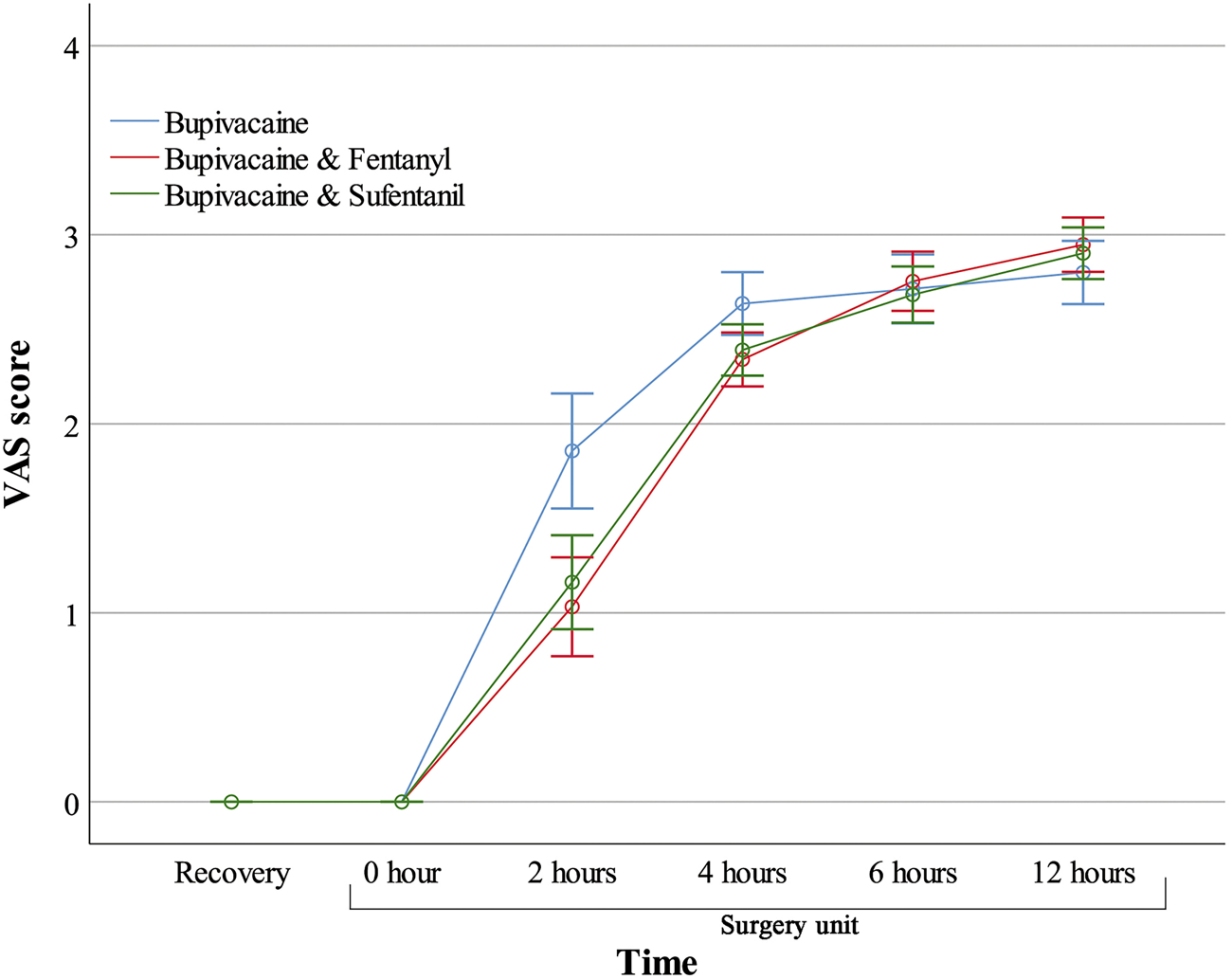
The mean of pain score between the three study groups at six time points was adjusted for the timing of the first morphine injection and the total morphine dosage administered.

Comparing the pain score mean in six‐time measurements showed that by adjusting for first‐time morphine injection, and the total number of times that morphine was given, the pain score mean in three groups increased over time, so that the pain was zero in all groups, on arrival in the recovery room, and when transferred to the surgical ward. However, after 2 h, the mean (SD) of pain score in Bupivacaine, Bupivacaine‐Fentanyl, and Bupivacaine‐Sufentanil increased to 1.73 (0.49), 1.10 (1.10), and 1.22 (1.25), respectively. This increase progressed to 4 h after surgery, so that the mean (SD) of pain scores in Bupivacaine, Bupivacaine‐Fentanyl, and Bupivacaine‐Sufentanil after 4 h were 2.61 (0.46), 2.36 (0.49), and 2.40 (0.58). However, after 4 h, the mean of pain scores was more similar in the three groups, so that in 12 h after surgery, mean (SD) of pain scores in Bupivacaine, Bupivacaine‐Fentanyl, and Bupivacaine‐Sufentanil were 2.76 (0.36), 2.97 (0.61), and 2.92 (0.56). This pattern was shown in Figure 2. As the result of within‐subject repeated measure ANOVA showed, a statistically significant difference in pain score was observed over time (*F* statistic = 388.981; df = 5880; *p* < 0.001).

#### 3.3.2 Over Time Pair‐Wise Comparisons

Bonferroni test results for pair‐wise comparisons at the different times showed that pain score at 2 h (difference: −1.350; *p* < 0.001), 4 h (difference: −2.46; *p* < 0.001), 6 h (difference: −2.72; *p* < 0.001) and 12 h (difference: −2.88; *p* < 0.001) after transferring to the operating room were more severe than the beginning time. Other pairwise comparisons are shown in Table 2.

**Table 2:** Result of the Bonferroni test for pair‐wise comparison of pain score over time.

Group	Difference^a^	*p* value
Recovery		
Beginning	0.0	—
< 2 h	−1.35	< 0.001*
< 4 h	−2.46	< 0.001*
< 6 h	−2.72	< 0.001*
< 12 h	−2.88	< 0.001*
Beginning		
< 2 h	−1.35	< 0.001*
< 4 h	−2.46	< 0.001*
< 6 h	−2.72	< 0.001*
< 12 h	−2.88	< 0.001*
2 h		
< 4 h	−1.11	< 0.001*
< 6 h	−1.37	< 0.001*
< 12 h	−1.53	< 0.001*
4 h		
< 6 h	−0.26	< 0.001*
< 12 h	−0.43	< 0.001*
6 h		
< 12 h	−0.17	< 0.001*

*Note:* All measurements were adjusted for first‐time morphine injection and the total number of times that morphine was given. *Significance level was considered as 0.05.

^a^ The difference was calculated as *before‐pain score minus after‐pain score*.

#### 3.3.3 Inter‐Participant Variability (Intervention Effect) on the Pain Scores

As shown in Table 3, by adjusting for first‐time morphine injection and the total number of times that morphine was given, the total mean score of pain in Bupivacaine, Bupivacaine‐Fentanyl, and Bupivacaine‐Sufentanil were 1.67 ± 0.05, 1.51 ± 0.09, and 1.52 ± 0.08, respectively. Results of between‐subjects in repeated measure ANOVA showed that pain score was significantly different between the three study groups (*F* statistic = 3.571; df = 2176; *p* < 0.001).

**Table 3:** Result of repeated‐measure ANOVA for comparing the pain score between three study groups over time.

Times	Group			Intra‐subject effect	Inter‐subject effect	Interaction
	Bupivacaine (*N* = 60)	Bupivacaine‐Fentanyl (*N* = 60)	Bupivacaine‐Sufentanil (*N* = 60)			
Recovery	0 ± 0	0 ± 0	0 ± 0	*F* = 388.981; df = 5880; *p* < 0.001*	*F* = 3.571; df = 2176; *p* = 0.030*	*F* = 4.253; df = 10,880; *p* < 0.001*
Surgery unit						
Beginning	0 ± 0	0 ± 0	0 ± 0			
2 h	1.73 ± 0.49	1.10 ± 1.10	1.22 ± 1.25			
4 h	2.61 ± 0.47	2.36 ± 0.49	2.40 ± 0.58			
6 h	2.67 ± 0.57	2.78 ± 0.54	2.70 ± 0.58			
12 h	2.76 ± 0.36	2.97 ± 0.61	2.92 ± 0.56			
Total	1.67 ± 0.05	1.51 ± 0.09	1.52 ± 0.08			

*Note:* The mean ± standard deviation of pain was presented at six points. *Significance level was considered as 0.05. All estimations were adjusted for first‐time morphine injection and the total number of times that morphine was given.

The result of the Bonferroni post hoc test showed that the pain score in Bupivacaine was statistically higher than Bupivacaine‐Fentanyl (difference: 0.156; *p* = 0.013) and Bupivacaine‐Sufentanil (difference: 0.145; *p* = 0.015). However, there was no difference between Bupivacaine‐Fentanyl with Bupivacaine‐Sufentanil (difference: −0.011; *p* = 0.819) (Table 4 and Figure 2).

**Table 4:** The result of the LSD test for multiple comparisons of pain scores between the three study groups.

Group	Group	Difference	*p* value
	Bupivacaine		
Bupivacaine	> Bupivacaine‐Fentanyl	0.156	0.013*
	> Bupivacaine‐Sufentanil	0.145	0.015*
	Bupivacaine‐Fentanyl		
Bupivacaine‐Fentanyl	= Bupivacaine‐Sufentanil	−0.011	0.819

*Note:* All estimations were adjusted for first‐time morphine injection and the total number of times that morphine was given. *Significance level was considered as 0.05.

#### 3.3.4 The Interaction Between the Intervention Time and Pain Score

The results showed a significant association between the intervention time and pain score (*F* statistic = 4.253; df = 10,880; *p* < 0.001). In particular, the pain score growth rate in the Bupivacaine group was significantly quicker than in the Bupivacaine‐Fentanyl and Bupivacaine‐Sufentanil groups, and these fluctuations were not affected by first‐time morphine injection and the total number of times that morphine was given (Table 3).

### 3.4 Comparison of Secondary Outcomes in Three Study Groups

**Figure 3:**
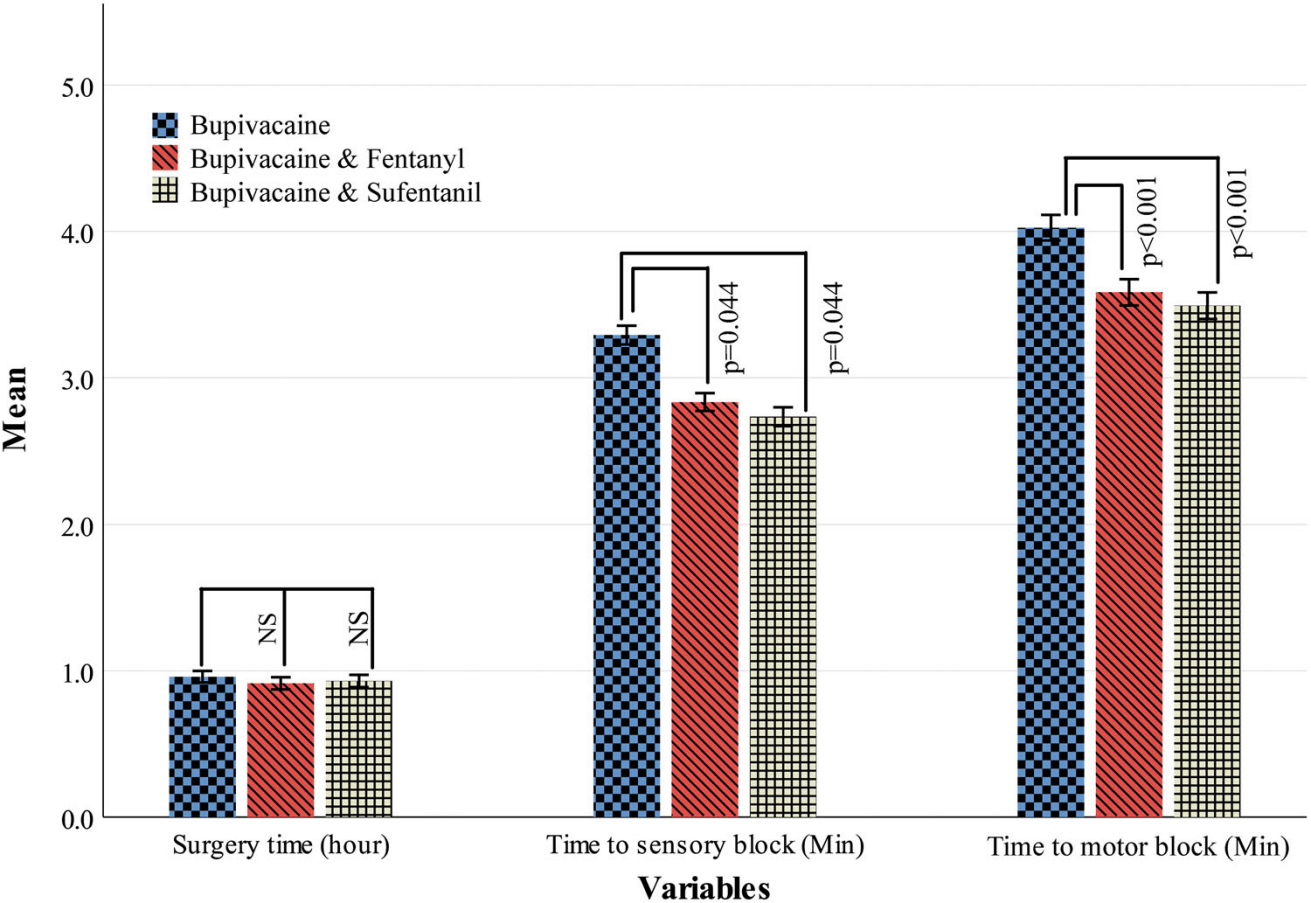
Pairwise comparison of secondary outcomes between the three study groups.

Results of the present study showed that the mean (SD) of surgery time (hours) was 0.96 (0.15) hours in the Bupivacaine group, 0.91 (0.16) hours in the Bupivacaine‐Fentanyl group, and 0.93 (0.16) hours in the Bupivacaine‐Sufentanil group, respectively. As the ANOVA test showed, surgery time was not statistically different in the three groups (*p* = 0.288). However, the mean (SD) of time (minutes) to sensory block to T6 in Bupivacaine 3.29 (0.25) minutes, Bupivacaine‐Fentanyl 2.83 (0.24) minutes, and Bupivacaine‐Sufentanil 2.73 (0.25) minutes was significantly different (*p* < 0.001). In addition, these values for the time to motor block were 4.03 (0.35) minutes, 3.58 (0.36) minutes, and 3.49 (0.36) minutes and were significantly different between the three study groups (*F* statistic = 40.83; df = 2176; *p* < 0.001). According to Figure 3, results from the Tukey post hoc test for pairwise comparison, the Bupivacaine group's mean motor block score was 0.441 min higher than that of the Bupivacaine‐Fentanyl group (*p* < 0.001) and 0.534 min higher than that of the Bupivacaine‐Sufentanil group (*p* < 0.001). The mean motor block scores for the Bupivacaine‐Fentanyl and Bupivacaine‐Sufentanil groups, however, did not differ significantly (*p* = 0.146).

### 3.5 Comparison of Side Effects Between the Three Groups

Table 5 shows the incidence of side effects in three study groups. Based on the Chi‐square test, and as shown in Table 5, there are no differences between the three groups for nausea (*p* = 0.158), vomiting (*p* = 0.862), pruritus (*p* = 0.814), and bradycardia (*p* = 0.718). Other side effects are also shown in Table 5.

**Table 5:** The incidence of side effects in three study groups.

Complication	*N* (%)			*p* value^a^
	Bupivacaine (*N* = 60)	Bupivacaine‐Fentanyl (*N* = 60)	Bupivacaine‐Sufentanil (*N* = 60)	
Nausea				
No	56 (93.3)	52 (86.7)	49 (81.7)	0.158
Yes	4 (6.7)	8 (13.3)	11 (18.3)	
Vomiting				
No	58 (96.7)	57 (95)	58 (96.7)	0.862
yes	2 (3.3)	3 (5)	2 (3.3)	
Pruritus				
No	59 (98.3)	58 (96.7)	58 (96.7)	0.814^b^
Yes	1 (1.7)	2 (3.3)	2 (3.3)	
Bradycardia				
No	57 (95)	55 (91.7)	55 (91.7)	0.718
Yes	3 (5)	5 (8.3)	5 (8.3)	
Hypotension				
No	50 (83.3)	39 (65)	42 (70)	0.066
Yes	10 (16.7)	21 (35)	18 (30)	

^a^ Calculated by the Chi‐square test.

^b^ Calculated by Fisher's exact test.

## 4 Discussion

The present results regarding pain and duration demonstrated that among the three intervention groups, Bupivacaine combined with Sufentanil exhibited superior performance from the first hour to 4 h post‐operation. Subsequently, Bupivacaine combined with Sufentanil and Bupivacaine combined with Fentanyl provided better pain control than Bupivacaine alone. However, although results showed some evidence of improved pain control in patients who received Bupivacaine combined with Sufentanil and Fentanyl, differences between groups did not meet conventional levels of statistical significance after 4 h post‐operation. Fentanyl and Sufentanil are synthetic opioid analgesics commonly utilized for pain management in general anesthesia during various surgical procedures [28].

Inguinal hernia repair is a common procedure performed across all age groups, and effective sedation and analgesia are crucial for its success. A short duration of sedation and quick recovery are preferred to minimize complications and reduce hospital stays [29]. The effectiveness of [30] postoperative sedation largely depends on the medications used during surgery. Sufentanil and Fentanyl are both potent analgesics that exhibit fewer side effects compared to traditional opioids [31]. However, Sufentanil is more potent than Fentanyl and has a shorter duration of action, as demonstrated in various studies. Research by Abbasnejad et al. [30] indicated that analgesia duration with Sufentanil for SA during inguinal hernia operations was longer than that with Fentanyl. Although Xia's [31] trial administered analgesia drugs systemically (by intravenous injection), Abbasnejad's [30] study administered analgesia drugs intrathecally; therefore, the injection strategy should not be disregarded. Since the duration of intrathecal action is likely more correlated with lipophilicity (i.e., the length of stay in the dorsal horn of the spine), these disparate approaches are significant.

The present study showed that Fentanyl and Sufentanil had similar analgesic effects after operation. In line with the present study, for other surgeries, Fentanyl and Sufentanil may not be different, and their effectiveness is the same. Lee et al. [15] demonstrated that in patients undergoing cesarean section, there was no significant difference in the duration of effective analgesia between Fentanyl and [32] Sufentanil administered via SA. Similarly, Farzi et al. [10] demonstrated that in patients following cesarean section with spinal anesthetic, the duration of analgesia was equivalent between the Fentanyl and Sufentanil groups. Those findings were also obtained in Duman et al.'s [32] study that the quality of SA in children undergoing inguinal hernia repair between Fentanyl and Sufentanil is the same. Based on our findings and the studies mentioned [10, 15, 32], we can say that adding Fentanyl and Sufentanil to SA offers good pain relief during surgery without major side effects, not just for hernia repair but also for other types of surgeries.

Our study found that using a specific anesthesia method resulted in side effects such as nausea, vomiting, pruritus, and bradycardia. However, the incidence of these side effects was similar across the three groups, and there was no significant difference among them, which corroborates findings from other studies [33, 34]. Weigl et al. [34] demonstrated that the incidence of postoperative nausea and vomiting in patients who received intrathecal fentanyl was comparable to those who received bupivacaine. Similarly, Akan et al. [33] reported no difference in postoperative nausea and vomiting between Sufentanil and bupivacaine. In contrast, a large systematic review and meta‐analysis by Fonseca et al. [35] also found that the use of Fentanyl and Sufentanil was associated with an increase in pruritus; however, our study did not observe this finding. This discrepancy may be attributed to differences in anesthetic regimens, surgical procedures, and perioperative care. The primary reason for the difference is likely related to the type of studies conducted. In Fonseca's [35] study, many of the included primary studies showed no association between the type of analgesia used and the occurrence of pruritus.

The present study revealed a statistically significant difference in the average time to motor block among the studied groups. Sufentanil combined with Bupivacaine demonstrated the shortest onset time, followed by Fentanyl with Bupivacaine, and finally Bupivacaine alone. While the absolute difference in onset time was less than 1 min—raising questions about its clinical significance—we believe this finding remains valuable for understanding the nuanced pharmacodynamic profiles of these drug combinations. In high‐turnover surgical settings or in patients where rapid onset is desirable, even modest reductions in onset time may contribute to improved workflow efficiency. Our results are consistent with those of Kuusniemi et al. [36], who also found that adding Fentanyl or Sufentanil to Bupivacaine accelerated motor block onset, though the differences diminished by 4 h postoperatively. This suggests that while the enhanced onset may be short‐lived, it could still offer practical benefits in specific clinical scenarios. Additionally, inter‐individual variability may also have influenced our results and deserves further investigation.

## 5 Limitations

This study focused on inguinal hernia surgeries, with patients exhibiting diverse race, occupation, and age characteristics. Given the limitations observed in comparable studies, further research with larger sample sizes, longer durations, and extended post‐operative follow‐ups is advisable. Additionally, it is recommended to investigate the effects of these three drug groups in various surgical contexts, including orthopedics, cardiology, and pulmonology. Furthermore, exploring different dosage regimens would contribute to a more comprehensive understanding of their efficacy. Finally, although urinary retention is a side effect of both spinal blockade with bupivacaine and intrathecal opioids, we did not assess it as an outcome in the present study due to certain infrastructure limitations that occurred during the study, even though our interventions may have contributed to its incidence.

## 6 Conclusion

The pain‐reducing effects of combining Sufentanil and Fentanyl with Bupivacaine are significantly greater than using Bupivacaine alone. The findings indicate that by adjusting the timing of the first morphine injection and the total morphine dosage, the superiority of the combination of Sufentanil and Fentanyl was evident for up to 4 h post‐operation. However, by 6 and 12 h after surgery, these differences diminished. The addition of Sufentanil and Fentanyl not only effectively reduced time to sensory block but also shortened the time to motor block. This suggests that the combined use of these agents is more effective in managing intraoperative pain than Bupivacaine alone, with effects persisting for up to 12 h after the procedure.

## Author Contributions

**Haniyeh Soleimany:** conceptualization, writing – original draft, investigation, project administration. **Hamed Tavan:** investigation, project administration, data curation, resources, writing – original draft. **Ebrahim Khalighi:** writing – original draft, writing – review and editing, investigation, conceptualization, data curation, supervision, resources, project administration. **Reza Pakzad:** conceptualization, investigation, writing – original draft, writing – review and editing, visualization, methodology, software, formal analysis, project administration, supervision.

## Conflicts of Interest

The authors declare no conflicts of interest.

## Transparency Statement

The lead authors Ebrahim Khalighi and Reza Pakzad affirm that this manuscript is an honest, accurate, and transparent account of the study being reported; that no important aspects of the study have been omitted; and that any discrepancies from the study as planned (and, if relevant, registered) have been explained.

## Supporting information


**Appendix:** CONSORT 2010 checklist of information to include when reporting a randomised trial*.

## Data Availability

The data that support the findings of this study are available from the corresponding author upon reasonable request.
